# *Xylella fastidiosa* Differentially Accumulates Mineral Elements in Biofilm and Planktonic Cells

**DOI:** 10.1371/journal.pone.0054936

**Published:** 2013-01-22

**Authors:** Paul A. Cobine, Luisa F. Cruz, Fernando Navarrete, Daniel Duncan, Melissa Tygart, Leonardo De La Fuente

**Affiliations:** 1 Department of Biological Sciences, Auburn University, Auburn, Alabama, United States of America; 2 Department of Entomology and Plant Pathology, Auburn University, Auburn, Alabama, United States of America; University of Wisconsin-Milwaukee, United States of America

## Abstract

*Xylella fastidiosa* is a bacterial plant pathogen that infects numerous plant hosts. Disease develops when the bacterium colonizes the xylem vessels and forms a biofilm. Inductively coupled plasma optical emission spectroscopy was used to examine the mineral element content of this pathogen in biofilm and planktonic states. Significant accumulations of copper (30-fold), manganese (6-fold), zinc (5-fold), calcium (2-fold) and potassium (2-fold) in the biofilm compared to planktonic cells were observed. Other mineral elements such as sodium, magnesium and iron did not significantly differ between biofilm and planktonic cells. The distribution of mineral elements in the planktonic cells loosely mirrors the media composition; however the unique mineral element distribution in biofilm suggests specific mechanisms of accumulation from the media. A cell-to-surface attachment assay shows that addition of 50 to 100 µM Cu to standard *X. fastidiosa* media increases biofilm, while higher concentrations (>200 µM) slow cell growth and prevent biofilm formation. Moreover cell-to-surface attachment was blocked by specific chelation of copper. Growth of *X. fastidiosa* in microfluidic chambers under flow conditions showed that addition of 50 µM Cu to the media accelerated attachment and aggregation, while 400 µM prevented this process. Supplementation of standard media with Mn showed increased biofilm formation and cell-to-cell attachment. In contrast, while the biofilm accumulated Zn, supplementation to the media with this element caused inhibited growth of planktonic cells and impaired biofilm formation. Collectively these data suggest roles for these minerals in attachment and biofilm formation and therefore the virulence of this pathogen.

## Introduction

Metals are required for the myriad of functions within all cells with almost half of all enzymes requiring a metal cofactor [1] Trace elements such as copper, manganese, zinc and iron are used as cofactors in proteins for structural and/or enzymatic processes. Redox active Cu and Fe cofactors are critical for photosynthesis in plants, and for the utilization of oxygen in eukaryotes. These metals are under tight homeostatic control because although required they also have the potential to be toxic at high concentrations. Multi-cellular organisms need to regulate the concentration and distribution of all metals at a cellular level to provide sufficient metals for the developing tissues [2]. The metal quota of an organism is defined as the total concentration of mineral elements required for cell viability [3]. The composition of the elements required for survival for bacteria is essentially the same as that required for a plant or human host [3]. The similarities in requirements mean that microbial pathogens face a unique challenge for survival inside the host organism, where they need to recruit the same essential cofactors and metabolites that the host carefully guards. Therefore pathogens must develop strategies to bypass host defenses and to ‘outcompete’ the host for certain elements.

*Xylella fastidiosa* is a Gram-negative bacterial plant pathogen that is found in more than 30 different plant hosts, causing disease in economically important plants including citrus and grapes [4], [5]. After infection via leafhopper insects the bacterium colonizes the xylem vessels and forms biofilm aggregates that block the passage of water and nutrients from the roots to the leaves [6], [7]. Visual symptoms of *X. fastidiosa* infection include leaf scorch, dwarfing, matchsticking and chlorotic spots [6]. Scorched tissues in infected leaves are water deficient [8] while some research argues that water-deficit symptoms in grapes differ from symptoms caused by *X. fastidiosa* infection [9], [10]. Regardless of the rate of transpiration to symptomatic leaves, minerals elements are important components of xylem sap, and are potential nutrients affected by, or affecting *X. fastidiosa* infection.

A number of *in vitro* and *in planta* experiments have revealed important roles for mineral elements in *X. fastidiosa*. The growth rate of the planktonic *X. fastidiosa* in xylem fluid has been correlated to the concentration of inorganic ions such as Cu, Mg, P and Zn, among other components such as amino acids and citric acid [11]. Iron regulates the expression of several *X. fastidiosa* pathogenicity factors e.g. type IV pilus and bacteriocins [12] and studies examining the toxicity of copper suggested that biofilm cells are more resistant to this metal than planktonic cells [13]. Additionally Ca can act as an ionic stabilizer of the biofilm matrix [14], [15], [16]. *Xylella fastidiosa* biofilm *in planta* has increased concentration of divalent cations suggesting a model where Ca and Mg bridge between the negatively-charged xylem vessels and the negatively-charged bacterial exopolysaccharides (EPS) [15].

In a recent investigation we demonstrated that *in vitro* supplementation of the standard growth medium with calcium concentrations closer to those found in xylem sap of susceptible hosts (in grape approximately 2–3 mM) promoted *X. fastidiosa* enhanced adhesion, biofilm formation and twitching motility, all critical virulence determinants [17]. Intracellular concentrations and metabolic activity were required for triggering Ca-enhanced attachment, and increases in total Ca concentrations were correlated to thicker biofilms and increased cell-to-cell aggregation. Therefore our results demonstrated a role for Ca as an intracellular trigger for aggregation and adhesion and supported the previous role as extracellular ‘glue’ in biofilms [15], [17]. In the communication by Cruz et al., initial experiments testing the effect of supplementation of mineral elements on attachment of *X. fastidiosa* [17], indicated that other specific elements promote or prevent biofilm formation. However that study did not address the levels of mineral elements found in the biofilm compared to planktonic cells. Based on previous observations, we hypothesized that mineral elements differentially accumulated in *X. fastidiosa* biofilm are required for cell attachment and aggregation. To test this hypothesis we measured the ionome in the biofilm and planktonic states. The ionome is defined as a snapshot of the total mineral element content of cells regardless of chemical form [18], [19]. We then tested the significance of mineral element accumulation in biofilm using *in vitro* assays of attachment and aggregation. Determining the ionome of *X. fastidiosa* is a step towards understanding the roles of mineral elements in virulence.

## Experimental

### Bacterial strains and culture conditions

*Xylella fastidiosa* subsp. *fastidiosa* strain Temecula (ATCC 700964) was used in this study and was grown in PD2 [20] liquid medium at 28°C. Stocks of *X. fastidiosa* cultures were stored in PD2 broth plus 20% glycerol at −80°C.

### Biofilm quantification in 96-well plates

Biofilm formation by *X. fastidiosa* was assessed by a crystal violet (CV)-based assay according to Cruz et al. [17] with cells grown in PD2 supplemented with metals and chelators at different concentrations. PD2 was supplemented with CuSO_4,_ MnCl_2_, ZnCl_2_ in a range of concentration from 0.01 mM to 2.5 mM. To limit Cu availability the cell-impermeant copper chelator bathocuproine sulfate (BCS) was added to PD2 media in a range of concentration from 0.01 mM to 0.15 mM. For all experiments using CV assay, each plate included a control row (n = 12) of cells grown in non-supplemented PD2. All experiments were replicated at least three times.

### Ionome quantification

Glass culture tubes containing 5 ml of liquid cultures of *X. fastidiosa* in PD2 medium were incubated with shaking (150 rpm) at 28°C for 7 days before harvesting planktonic and biofilm cells. Planktonic samples were removed from the liquid by centrifugation 5 minutes at 3500× g then washed twice in ultra-pure water (MilliQ, Millipore, MA) and once in 0.05 M EDTA to remove non-specifically associated mineral elements. The biofilm cells attached to the glass surface at the air-liquid interphase were removed by vortexing the culture tubes with ultra-pure water. Cells were then collected by centrifugation and washed as per the planktonic cells.

Samples were digested for at least 1 hour at 100°C in 200 µL of metal-free concentrated nitric acid (OPTIMA, Fisher Scientific). After dilution with ultra-pure, metal-free water (MilliQ, Millipore, MA) and centrifugation at 13,000× g to remove any particulates, samples were analyzed by Inductively Coupled Plasma with Optical Emission Spectroscopy (ICP-OES, Perkin Elmer 7100 DV, Waltham, MA) with simultaneous measurement of B, Ca, Co, Cu, Fe, K, Mg, Mn, Mo, Na, Ni, S, Zn. Mineral element concentrations were determined by comparing emission intensities to a standard curve created from certified standards (SPEXCertiprep, Metuchen, NJ). Standard curves were confirmed by re-analysis of standard solutions diluted in a matrix equivalent to the sample as required. Individual readings (average of two intensity measurements) and repeated analysis of individual samples showed less the 5% variation.

Each ionome experiment consisted of 10 individual cultures grown in a set, and this experiment was repeated independently four times to generate the average ionome. Sulfur concentration was assessed as a function of cell number measured by optical density (OD_600_) in 40 independent cultures to establish a correlation. The linear correlation (data not shown: for planktonic (y = 2.2457×–0.5508, R^2^ = 0.87) and biofilm (y = 2.6508×–0.0484 R^2^ = 0.93)) suggests sulfur can be used as an internal control for potential loses occurring in the preparation or reading of the samples.

### Assessment of biofilm formation in microfluidic chambers

The formation of biofilm was evaluated in microfluidic chambers in PD2 and PD2 supplemented with either 50 or 400 µM CuSO_4_. The fabrication of the chambers was performed as previously described [21].A two parallel channels chamber design was used for the experiments (80 µm wide by 3.7 cm long by 50 µm deep), each channel has two inlets to allow the separate entry of media and bacteria and an outlet to allow media to flow out the other end. For biofilm assessment eight-day-old bacterial cultures were scraped from PD2, suspended in PD2 or PD2 + Cu (50 or 400 µM). Bacterial suspensions were introduced into the microfluidic chamber through tubing connected to the chamber. The chamber flow controlled by an automated syringe pump (Pico Plus; Harvard Apparatus, Holliston, MA) was kept at 0.25 µl min^−1^. Microfluidic chamber was mounted onto a Nikon Eclipse Ti inverted microscope (Nikon, Melville, NY) and observations were made at 40X with phase-contrast optics. Time lapse microscope images were acquired every 2 min during 5 days with a Nikon DS-Q1 digital camera (Nikon, Melville, NY) controlled by NIS-Elements software (Nikon, Melville, NY).

### Bacterial cell-to-cell aggregation assessment using VIS-spectroscopy

Bacterial aggregation was examined in *X. fastidiosa* from 5 ml of PD2 liquid cultures incubated with shaking at 28°C for 7 days. Cells were harvested and suspended in ultra-pure water. Cell-to-cell aggregation was established via “settling rate” [17] using a UV-Vis spectrophotometer (Model 2450, Shimadzu scientific instruments, Columbia, MD). Cell suspension was homogenized vigorously by pipetting and placed in the spectrophotometer where OD_600_ was continuously measured for 1 minute. The “settling rate” was calculated as the slope of the linear portion of decreasing change in OD_600_ over time.

### Collection of xylem sap

Xylem fluid (sap) was collected from six different grape varieties growing in Montaluce Winery and Vineyards near Dahlonega, Georgia, using a protocol previously described [22]. One to three vines per variety (∼3 years old) were tapped, including *Vitis* ‘Vidal’ and ‘Petite Vidal’, and *Vitis vinifera* ‘Cabernet Sauvignon’, ‘Merlot’, ‘Malbec’, and ‘Sangiovese’. Collection occurred in April 2010 and was performed by cutting canes and allowing the xylem contents to drip into 50 ml conical tubes for 1–2 h. Samples of the same variety were pooled, and the amount of sap collected per variety ranged from 2 to 19 ml. Samples were kept cold in an iced cooler until return to the laboratory, where they were filter-sterilized using a 0.22 µm-membrane filter, and frozen at −80°C for further use. For mineral element composition analysis, samples were defrosted and 1 ml used for ICP-OES analysis. No specific permits were required for the described field studies. Montaluce Winery via Oliver Asberger provided permission to sample and granted access to privately owned land.

### Statistical analysis

Data from the ionome characterization and attachment assay were analyzed by two-tailed Student's t-test using the Microsoft Excel for Mac 2011 software and values of *p*<0.05 were considered significant.

## Results and Discussion

### Ionome differences between biofilm and planktonic cells

**Figure 1:**
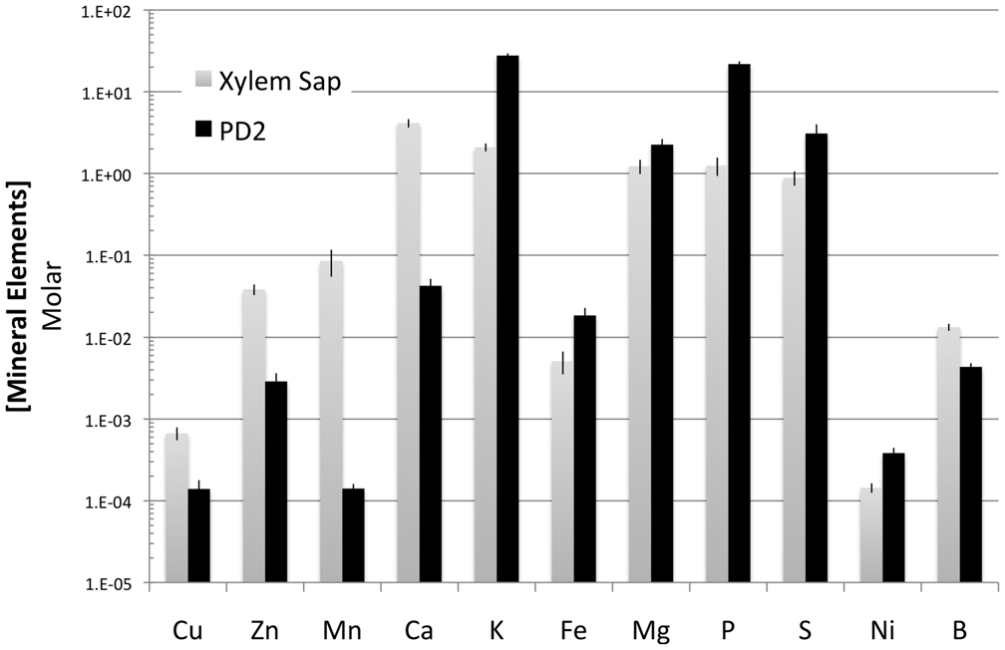
Ionome of *X. fastidiosa* synthetic medium (PD2) versus field-collected grape xylem sap. PD2 batches (n = 4) and xylem sap from different grape varieties (n = 6) were analyzed via ICP-OES. Values are presented as molar concentrations and represent average and standard errors of the mean (SEM).

**Figure 2:**
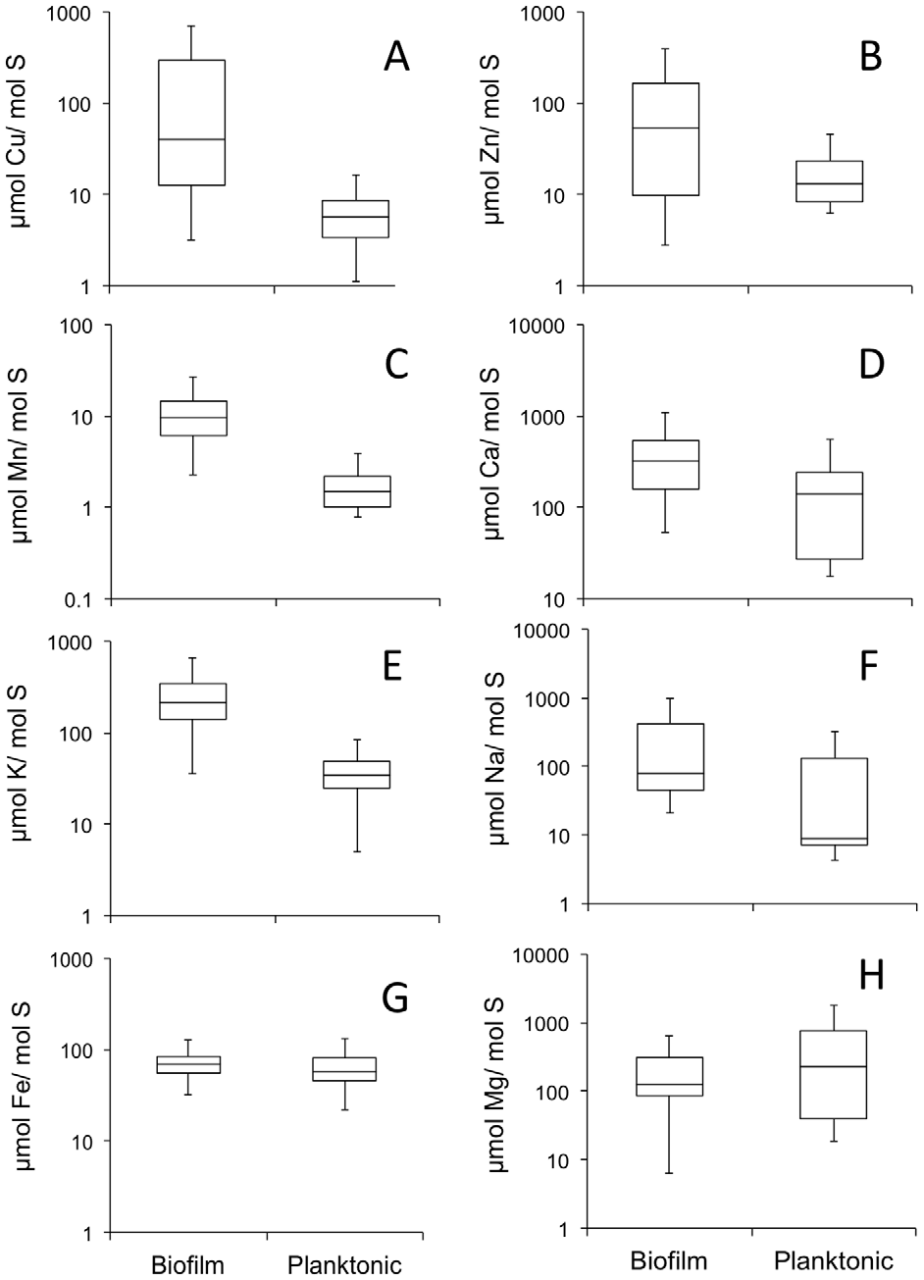
Box-plot representation of mineral element concentrations in *X. fastidiosa* biofilm or planktonic cells. The box represents the interquartile range for the samples and the whiskers the total range (n = 40) A) Cu B) Zn C) Mn, D) Ca, E) K, F) Na, G) Fe, and H) Mg. Significant differences are observed in Cu, Zn, Mn, Ca and K (t-test *p*<0.05, see Table 1).

As a comparison between the media used for bacterial growth *in vitro* and the natural environmental conditions where the bacteria survives, we measured the average mineral element content of PD2 media compared to the concentrations of mineral elements in xylem sap of susceptible grape varieties. We observed that standard PD2 media compared to xylem sap has relatively low levels of Cu, Zn, Mn and Ca (Fig. 1). On the other hand, K and P were more concentrated in PD2 than in xylem sap, while Fe, Mg, S, Ni and B had similar levels in both media types (Fig. 1). Culturing *X. fastidiosa* in xylem sap improves biofilm formation [22], therefore we put an emphasis on those elements that are more concentrated in sap. When *Xylella fastidiosa* cultures were analyzed, nine mineral elements (Ca, Cu, Fe, Mg, Mn, Na, K, Zn, S) were reliably detected in biofilm and planktonic cells under our conditions, while B, Co, Mo, Ni were undetectable on the amount of cells available for analysis. We used sulfur content as a denominator in these studies that could account for losses in the digest or internal errors in the ICP-OES readings. Significant changes were observed in the levels of multiple mineral elements in comparisons of biofilm compared to planktonic cells (Table 1). Copper was found at 30-fold higher concentrations (*p*<0.001), Mn found at 6-fold higher concentrations (*p* = 0.02) and Zn found at 5-fold higher concentrations (*p*<0.001), while Ca (*p* = 0.03) and K (*p* = 0.03) were at 2-fold higher concentrations in biofilm versus planktonic cells (Table 1). With the exception of K, all these elements were less concentrated in PD2 than in grape xylem sap (Fig. 1). No significant differences were observed for Fe (*p* = 0.13), Na (*p* = 0.07), and Mg (*p* = 0.13) comparing planktonic and biofilm cells (Table 1). To show the variability in these data (n = 40) the range of mineral elements concentrations detected and the median values are shown in box plots (Fig. 2). The relative distribution of mineral elements in planktonic cells Mg>Ca>Fe>Zn>Cu>Mn is identical to that found in PD2 media, however biofilm undergo significant changes that creates a unique profile Ca = Cu>Zn>Mg>Fe>Mn.

**Table 1:** Mineral element concentration in *Xylella fastidiosa* in the planktonic and biofilm physiological states.

	Planktonic	Biofilm	Biofilm vs. Planktonic	Fold Difference^3^
	Mean ± SEM^1^	Mean ± SEM	*p*-value^2^	
	(µmol/mol S)	(µmol/mol S)		
Cu	6.6±0.8	208±47	<0.0001	30
Zn	19±3	97±19	0.0002	5
Mn	4±1	23±8	0.02	6
Ca	218±61	491±96	0.03	2
K	90±21	230±24	0.03	2
Na	113±35	481±134	0.07	4
Fe	66±6	86±10	0.13	1
Mg	690±213	261±49	0.13	0.4

1. SEM =  Standard error of the mean.

2. *p*-values <0.05 indicate significant difference between concentration of the element in biofilm versus planktonic states, according to Student's t-test.

3. Fold differences represent mean value for biofilm divided by mean value for planktonic cells.

The specific minerals found accumulating in biofilm compared to planktonic cells (Cu, Zn, Mn and Ca) are also lower in PD2 media compared to xylem sap (Fig. 1). So we focused on the potential roles on cell attachment and biofilm formation of Cu, Zn, and Mn. Calcium was also accumulated in the biofilm compared to planktonic cells (Table 1). Our previous study [17] showed a role for calcium in aggregation and attachment of *X. fastidiosa*. This suggested that the other mineral elements that accumulated in the biofilm compared to planktonic cells might also enhance, or be required for, different aspects of aggregation and attachment. Since calcium was the focus of our previous work [17], it will not be discussed further here. Also even though a 2-fold accumulation was observed in biofilm for K we did not pursue this element as it did not show any clear positive or negative affects on biofilm formation (data not shown and [17]).

### Copper and biofilm

**Figure 3:**
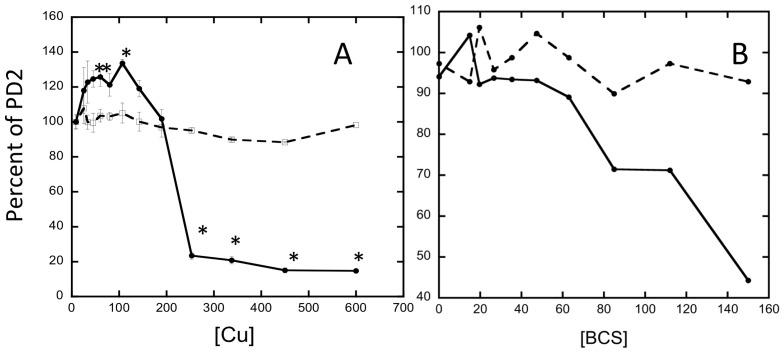
Quantification of attachment using crystal violet detection assay with variable Cu concentrations. Biofilm is assessed using absorbance at 600 nm of crystal violet staining after washing of the microtiter plate. Biofilm fraction is compared to the average biofilm content in PD2 media. A) Cu added up to 200 µM resulted in increased surface attachment, while attachment decreased when 200–600 µM Cu was added. Planktonic growth was unaffected by copper addition across the complete range 0–600 µM. The experiment was repeated three times, and each experiment contained three replicates. Asterisks indicate significant difference between copper supplemented and non-supplemented PD2 according to t-test (*p*<0.05). B) Copper chelation by the extracellular only chelator, bathocuproine sulfate, BCS, inhibits attachment at concentrations greater than ∼70 µM. The experiment was repeated three times, and one representative experiment is shown. In both panels solid lines represent biofilm quantification, while dotted line planktonic cells quantification. Copper or BCS concentrations are in µM.

Given the unusually high concentration of copper in biofilm we further investigated the role of copper in biofilm of *X. fastidiosa*. Copper is an essential and toxic element and therefore requires careful homeostatic control. Copper has been used in agriculture as an important antimicrobial and fungicide particularly in vineyards [23]. To interrogate the role of copper in biofilm formation we used a CV assay that assesses *X. fastidiosa* attachment to a surface. Using incremental additions of copper we observed a significant increase in biofilm at initial concentrations of copper (0 to 200 µM), while biofilm was reduced at higher concentrations (200 to 600 µM) (Fig. 3A). To complement these data we cultured *X. fastidiosa* in the presence of the cell-impermeant copper(I) chelator bathocuproine sulfate (BCS, extracellular only). This deprivation of copper resulted in decreased biofilm formation (Fig. 3B) suggesting that Cu acquisition, or association of extracellular Cu, is required for some aspect of forming or maintaining biofilm. We do note that the addition of BCS to grown cells did not affect cell-to-cell aggregation as measured by settling rate (data not shown). This suggests that if extracellular Cu is required for cell-to-cell aggregation, acting as a cross-linker, it is inaccessible to this chelator due to binding partners or redox state.

**Figure 4:**
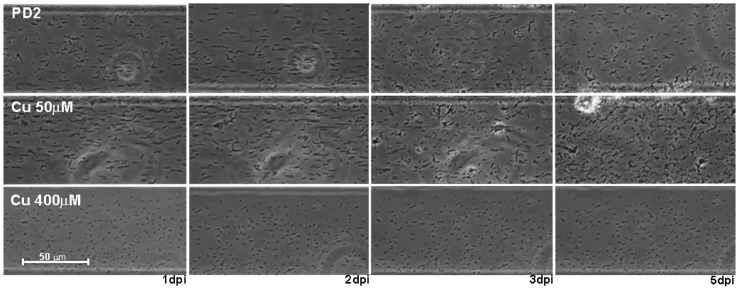
Evaluation of cell-to-cell aggregation of *X. fastidiosa* at different Cu concentrations. Time-lapse micrographs showing cell attachment and the formation of cell aggregates inside microfluidic chambers. Images were captured at 1, 2, 3, and 5 days post inoculation. PD2 or PD2 supplemented with 50 µM or 400 µM Cu was used as growth media.

These observations in CV assays were corroborated by visual observation of the attachment in microfluidic chambers. The addition of 50 µM copper to PD2 resulted in increased number of cells attached and cell aggregation occurred 1–2 days earlier compared to PD2 alone (Fig. 4). On the contrary no cell aggregates were formed when 400 µM Cu was added, possibly due to growth inhibition (Fig. 4).

**Figure 5:**
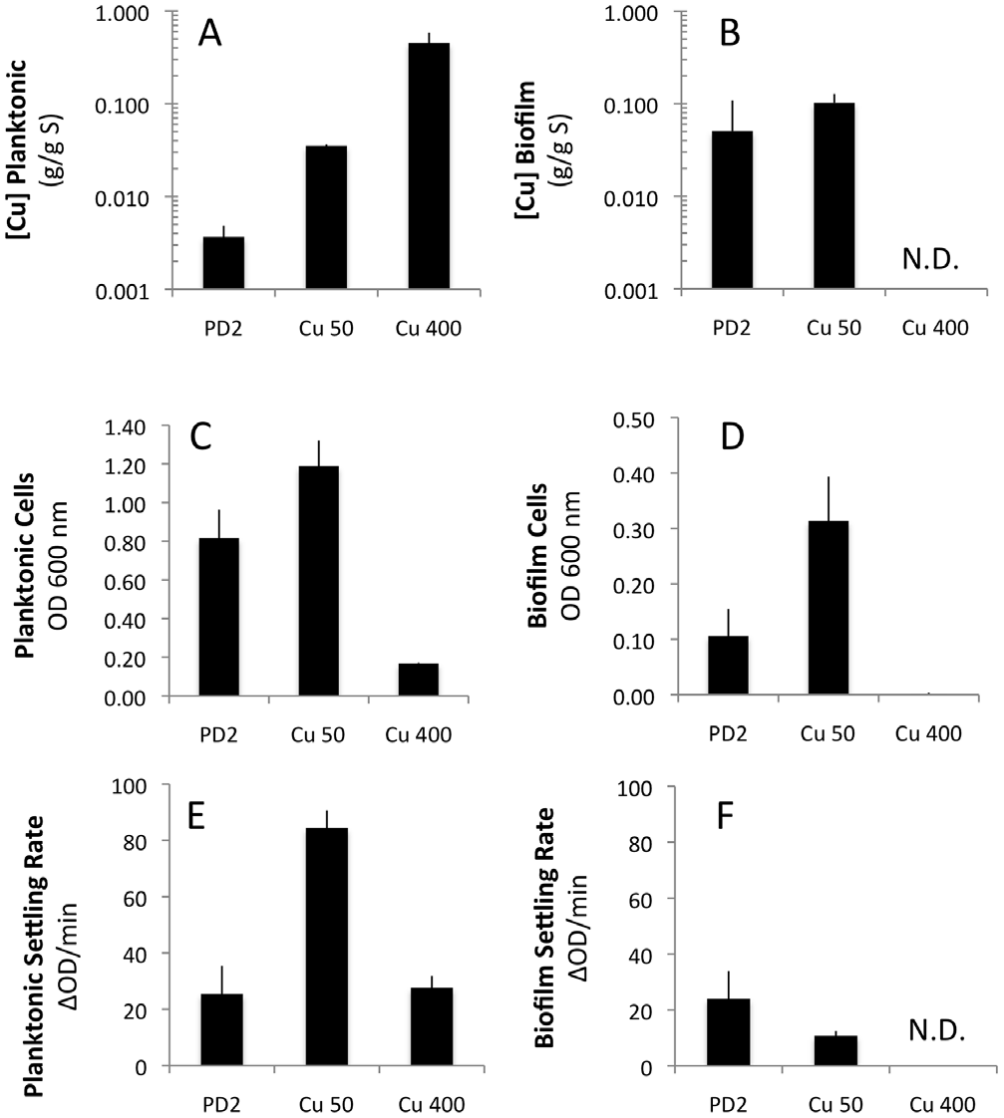
Cu addition to PD2 affects biofilm formation and cell-to-cell interactions. Cells grown in PD2 (n = 3) were compared to cells grown in 50 µM CuSO_4_ (n = 3) or 400 µM CuSO_4_ (n = 3). The planktonic cells (A, C, E) were separated from biofilm (B, D, F) and measured by ICP-OES for copper content (log 10 scale)(A, B), total optical density as a measure of cells in different states (C, D) and settling rate as a measure of cell-to-cell aggregation (E, F). N.D. not detectable due to low cell numbers. Data presented as mean and standard error of the mean.

To determine the levels of copper required to enhance attachment and aggregation or to induce toxicity we cultured *X. fastidiosa* in PD2 with supplemental addition of Cu. The concentrations used were based on observations from the crystal violet assay and microfluidic chambers (addition of 50 µM copper for enhanced attachment and 400 µM to prevent biofilm formation). *Xylella fastidiosa* grown in test tubes in PD2+Cu 50 µM accumulated 10-fold higher concentrations of copper in planktonic cells compared to PD2 alone, and accumulated only 1.5-fold higher concentrations of copper in biofilm compared to PD2 alone (Fig. 5A, B). It should be noted that in this experiment *X. fastidiosa* biofilm in PD2 had a 15-fold accumulation of copper compared to PD2 planktonic cells. The addition of 50 µM Cu improved the growth of planktonic cells and increased the level of biofilm formed when measured by optical density (Fig. 5C, D). In a cell-to-cell aggregation assay, measured by the settling rate of suspended cells, planktonic cells from the PD2+Cu 50 µM cultures had faster settling rates relative to untreated cells suggesting enhanced cell-to-cell aggregation (Fig. 5E). However, biofilm cells of PD2+Cu 50 µM showed decreased cell-to-cell aggregation compared to PD2 alone. The addition of 400 µM Cu inhibited growth of *X. fastidiosa*, decreased cell-to-cell aggregation (in planktonic cells compared to PD2+Cu 50 µM) and prevented biofilm formation (Fig. 5C, E). These results in test tubes confirmed the observations from microtiter plates (Fig. 3A, B), where cells form more cells aggregates (measured in CV assays) at an optimal Cu concentration (around 50 µM added Cu), but biofilm is inhibited at higher concentrations (400 µM).

The levels of copper in biofilm of untreated (PD2) cells did fluctuate significantly especially when compared to planktonic cells (Fig. 2A). Currently we cannot determine if the accumulation of copper happens before or after biofilm formation, and we see no obvious correlation between amount of copper in the biofilm and the amount of attached cells. Observations by others suggest that *X. fastidiosa* cells in the biofilm state are significantly more resistant to copper [13] and our data might suggest that the ability to accumulate copper in this physiological state may provide part of the protection. We speculate that the observation that copper does not dramatically increase in the biofilm of PD2+Cu 50 µM (Fig. 5B) may suggest that the level of copper accumulated is close to the maximum to allow for successful biofilm formation. Beyond this concentration presumably the loss of the ability to self-aggregate due to growth inhibition prevents biofilm formation.

Gene expression changes in *X. fastidiosa* induced by transient treatment of cells with sub-inhibitory and inhibitory concentrations of Cu were recently reported [24]. Genes implicated in biofilm formation, such as hemagglutinins that contribute to cell-to-cell adhesion [25], were induced by the sub-inhibitory levels of copper [24]. While these experimental conditions differ from ours, the increased availability of copper (PD2+Cu 50 µM) may induce similar increase in expression of hemagglutinins to facilitate the faster settling rate and increase in biofilm formation observed here.

Copper detoxification systems have been recognized previously as resistance genes in *Xanthomonas and Pseudomonas* sp. [26], [27], [28], [29]. In addition to anthropogenic challenges, pathogens must also evade the host defense mechanisms. Oxidative killing is a major route of pathogen clearance by the immune system and metals such as Cu, Fe, Zn and Mn are all used as prokaryotic antioxidant defenses [30]. Therefore specific metal requirements exist for sustained pathogenicity of various organisms. The pathogenesis of *Xanthomonas oryzae* the causal agent of bacterial blight in rice, has been linked to one susceptibility locus, Xa13 [31]. Xa13 encodes for a membrane protein that can affect the activity of Cu transporters that can remove Cu from the xylem [31]. The increased activity of the Cu transporters, leads to decreased Cu concentrations in the xylem and allows *X. oryzae* to multiple, resulting in severe bacterial blight [31]. The depletion of Cu in the xylem leading to increased pathogen survival suggests Cu is part of the host response to mediate pathogen removal.

Here we show an accumulation and a requirement for certain mineral elements in the formation of *X. fastidiosa* biofilm. It has been shown that *X. fastidiosa* ability to mediate cell-to-cell aggregation and therefore biofilm is a critical factor in the *in planta* survival [25]. Exopolysaccharides (EPS) production by *Erwinia amylovora*, the causative agent of fire blight in apples and pears, is increased during Cu stress [32]. The main constituents amylovoran and levan are able to bind Cu and therefore decrease its toxicity [32]. Therefore, it is possible that the accumulation of Cu in *X. fastidiosa* biofilm serves a similar protective role.

### Zinc, manganese and biofilm

**Figure 6:**
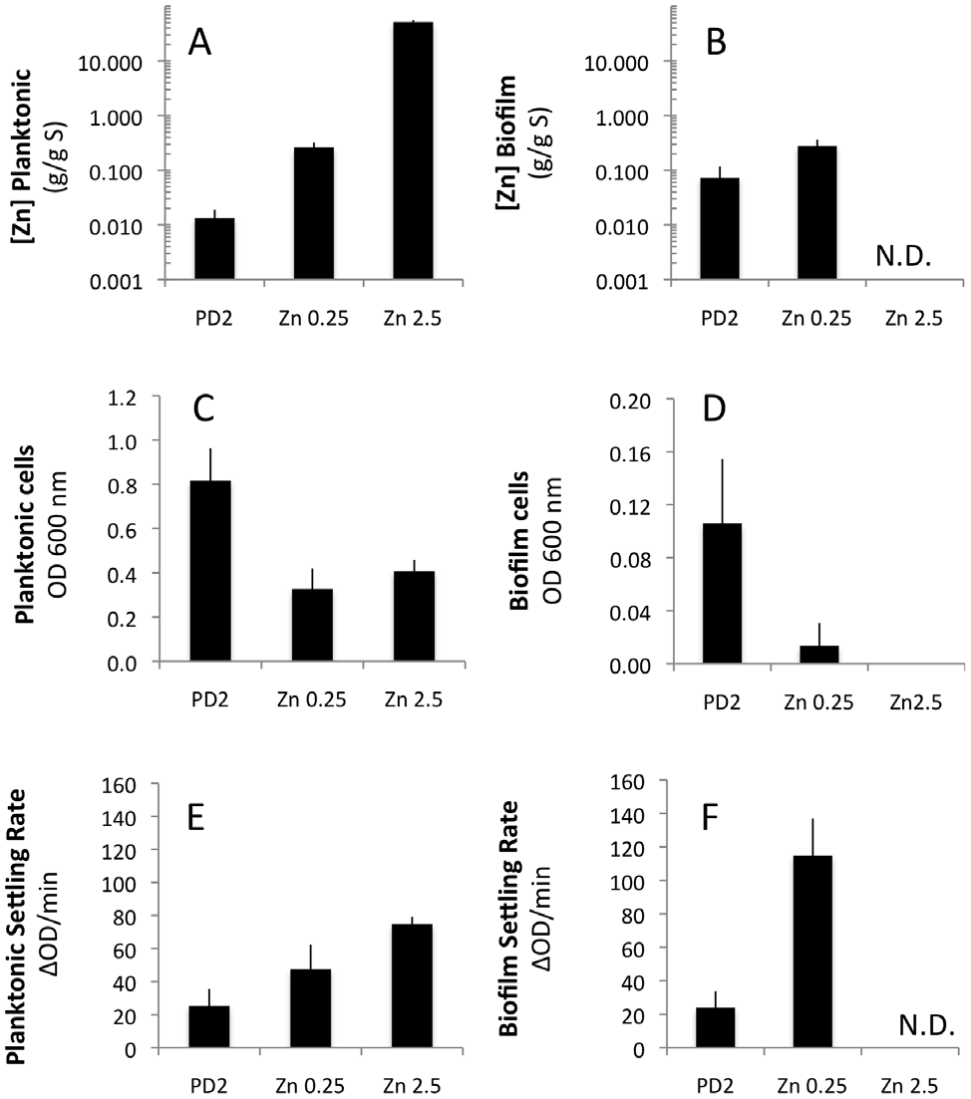
Zn addition to PD2 affects biofilm formation and cell-to-cell interactions. Cells grown in PD2 (n = 3) were compared to cells grown in 0.25 mM ZnCl (n = 3) or 2.5 mM ZnCl (n = 3). The planktonic cells (A, C, E) were separated from biofilm (B, D, F) and measured by ICP-OES for zinc content (log 10 scale)(A, B), total optical density as a measure of cells in different states (C, D) and settling rate as a measure of cell-to-cell aggregation (E, F). N.D. not detectable due to low cell numbers. Data presented as mean and standard error of the mean.

In addition to copper accumulation we observed that zinc and manganese levels were significantly higher in biofilm compared to planktonic cells. Zinc is a structural and catalytic cofactor in a variety of proteins including Zn-metalloproteases important in digesting plant components for the nutrition of the bacterium [33], [34], [35], [36]. Based on observations that supplemental Zn inhibited biofilm formation in microtiter plates [17] we chose to supplement PD2 media with zinc at 0.25 mM and at 2.5 mM. Zn added at 0.25 mM resulted in 20-fold accumulation in planktonic cells with a 50% reduction in growth and 90% decrease in biofilm while addition of 2.5 mM Zn (resulted in 5000-fold accumulation in planktonic cells) reduced biofilm to undetectable levels (Fig. 6A–D). The addition of 0.25 mM Zn to the media actually increased cell-to-cell aggregation in both planktonic and biofilm cells (Fig. 6E–F). Zinc has been implicated in EPS production in the plant pathogen *Xanthomonas campestris* pv. *campestris* with mutants lacking a Zn-regulated repressor producing less EPS and therefore decreased virulence [37]. Zn has also been linked to adhesion of bacteria to surfaces in animal pathogens but no reports of similar role in plant pathogen are known [38].

**Figure 7:**
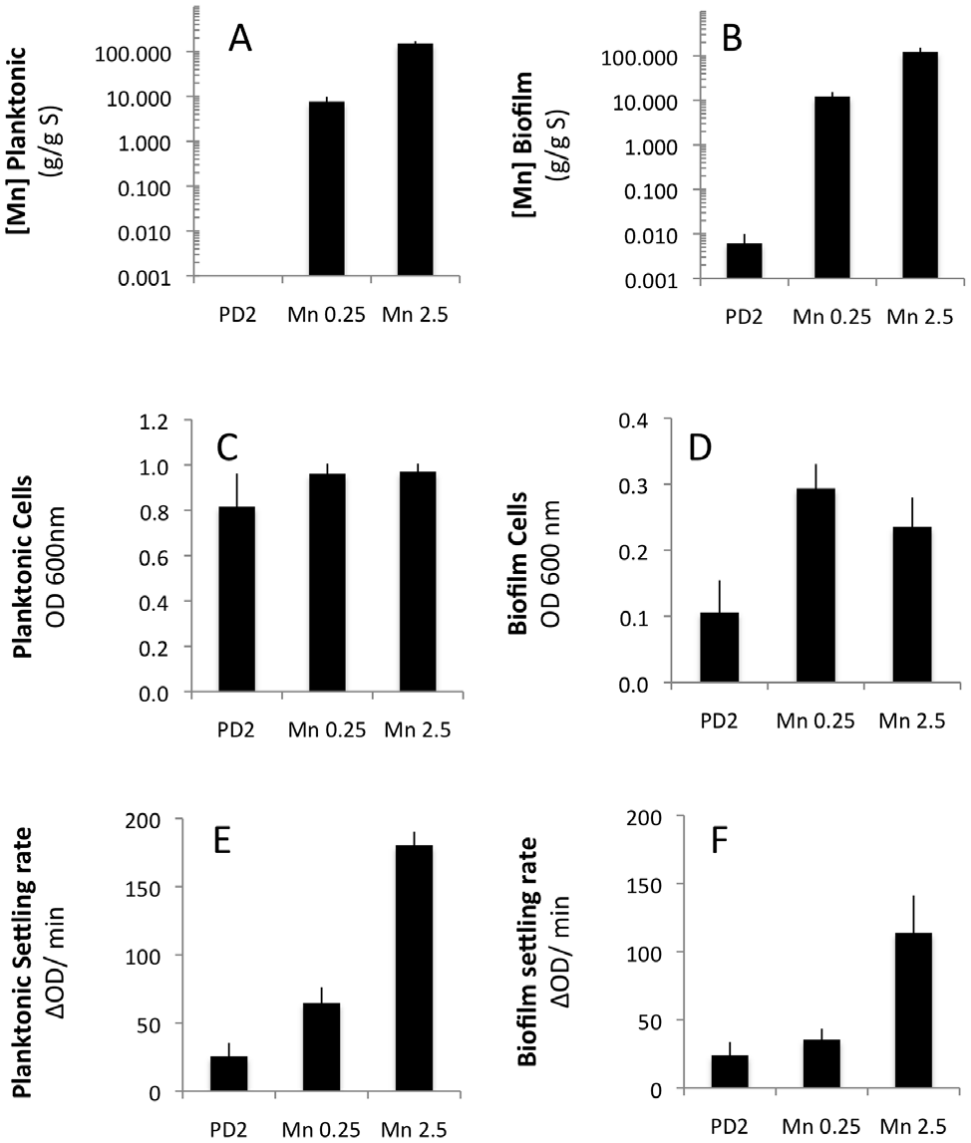
Mn addition to PD2 affects biofilm formation and cell-to-cell interactions. Cells grown in PD2 (n = 3) were compared to cells grown in 0.25 mM MnCl_2_ (n = 3) or 2.5 mM MnCl_2_ (n = 3). The planktonic cells (A, C, E) were separated from biofilm (B, D, F) and measured by ICP-OES for manganese content (log 10 scale)(A, B), total optical density as a measure of cells in different states (C, D) and settling rate as a measure of cell-to-cell aggregation (E, F). Data presented as mean and standard error of the mean.

Manganese like other mineral elements has been linked to EPS production adhesion and biofilm organization but these studies have been limited [39], [40]. Manganese has most commonly been linked to virulence by its role as a protectant against oxidative stress [41]. Manganese is a cofactor for many enzymes required for protection of the pathogen against oxidative stress, a host defense mechanism. *X. fastidiosa* infection in *Arabidopsis thaliana* induces gene expression of host oxidative stress protection [42]. Manganese levels increase in biofilm approximately 6-fold (Table 1). As expected, the addition of Mn to our test tube cultures did result in significant increases in the levels of Mn associated with both planktonic and biofilm cells, 3–4 orders of magnitude higher (Fig. 7A, B). These accumulations resulted in an increase in biofilm and cell-to-cell aggregation rates (Fig. 7C–F). The large accumulation may be consistent with a non-proteinaceous accumulation similar to that observed for *Neisseria* sp. isolates that produce Mn-oxide that is required for virulence to protect against oxidative stress [30], [43], [44]. By analogy we speculate that either proteinaceous or non-proteinaceous Mn may be important for protection of the *X. fastidiosa* in biofilm when in the xylem of the host plant.

## Conclusions

Pathogens must adapt to changing environments as they move from their reservoir to a host. A number of animal and plant pathogens induce large scale transcriptional remodeling in metal-related genes during the initial infection. Transcriptional activation of iron acquisition systems in pathogenic bacteria is often associated with establishment of infection in the host [45], [46]. These changes in transcription reflect the need to capture essential elements from the host. Some pathogens even cause metal remodeling of the host to enhance survival [31]. For some plant pathogens these changes may be in part a response of protection due to the widespread use of metal-based antimicrobials.

Further investigations will be required to identify the exact molecular roles of these metals in the pathogen-host interaction. This study reinforces the roles of mineral elements have in aggregation and biofilm formation, and therefore roles in establishment of infection and may suggest strategies for the design of treatments for *X. fastidiosa* infected crops based on inducing changes in homeostasis of these elements.
